# Normal Anti-Thyroid Peroxidase Antibody (TPO-Ab) Titers and Active Arterial Wall Thickening among Euthyroid Individuals: A Prospective Study

**DOI:** 10.3390/jcm11030521

**Published:** 2022-01-20

**Authors:** Yuji Shimizu, Shin-Ya Kawashiri, Yuko Noguchi, Seiko Nakamichi, Yasuhiro Nagata, Naomi Hayashida, Takahiro Maeda

**Affiliations:** 1 Department of General Medicine, Nagasaki University Graduate School of Biomedical Sciences, Nagasaki 852-8501, Japan; seiko-n@nagasaki-u.ac.jp (S.N.); tmaeda@nagasaki-u.ac.jp (T.M.); 2 Department of Cardiovascular Disease Prevention, Osaka Center for Cancer and Cardiovascular Diseases Prevention, Osaka 536-0025, Japan; 3 Department of Community Medicine, Nagasaki University Graduate School of Biomedical Sciences, Nagasaki 852-8523, Japan; shin-ya@nagasaki-u.ac.jp (S.-Y.K.); y-noguti@nagasaki-u.ac.jp (Y.N.); ynagata1961@nagasaki-u.ac.jp (Y.N.); 4 Leading Medical Research Core Unit, Nagasaki University Graduate School of Biomedical Sciences, Nagasaki 852-8523, Japan; naomin@nagasaki-u.ac.jp; 5 Nagasaki University Health Center, Nagasaki 852-8521, Japan; 6 Division of Promotion of Collaborative Research on Radiation and Environmental Health Effects, Atomic Bomb Disease Institute, Nagasaki University, Nagasaki 852-8523, Japan

**Keywords:** TPO-Ab, thyroid peroxidase, CIMT, atherosclerosis, active arterial wall thickening, yearly progression, euthyroid

## Abstract

Among euthyroid individuals, having an anti-thyroid peroxidase antibody (TPO-Ab) titer in the normal range (negative) is positively associated with atherosclerosis as evaluated based on carotid intima-media thickness (CIMT). Atherosclerosis is an established risk factor for cardiovascular disease, but no significant association between yearly progression in CIMT and cardiovascular disease has been reported. Therefore, clarifying the association between having a TPO-Ab titer in the normal range and yearly progression in CIMT (i.e., active arterial wall thickening) among euthyroid individuals could help inform strategies for preventing cardiovascular disease. We conducted a prospective study of 1069 Japanese subjects with free triiodothyronine and free thyroxine levels within the normal range. Having a TPO-Ab titer in the normal range was significantly positively associated with baseline atherosclerosis and significantly inversely associated with active arterial wall thickening. After adjusting for known confounding factors, the adjusted odds ratio (OR) and 95% confidence interval (CI) of log (TPO-Ab titer) for baseline atherosclerosis and active arterial wall thickening was 2.16 (1.07, 4.35) and 0.59 (0.37, 0.93), respectively. Since progression in CIMT is a process of aggressive endothelial repair, deficient endothelial repair inhibits active arterial wall thickening. Therefore, high–normal TPO-Ab titers might induce a deficiency in endothelial repair.

## 1. Introduction

Anti-thyroid peroxidase antibodies (TPO-Abs) are known to cause autoimmune thyroiditis. A previous study with three years follow-up of rheumatoid arthritis patients reported a larger carotid intima-media thickness (CIMT) progression in patients TPO-Ab positive than in those TPO-Ab negative. This study also found that TSH levels were higher in TPO-Ab positive patients than in TPO-Ab negative patients [1].

Subclinical hypothyroidism, defined as increased serum thyroid-stimulating hormone (TSH) concentrations and normal serum free thyroxine levels (free T4), is reported to be positively associated with carotid atherosclerosis [2]. Therefore, thyroid function could influence the association between TPO-Ab and the progression of atherosclerosis evaluated by CIMT. However, our previous cross-sectional study found a significant positive association between normal TPO-Ab titers (negative) and carotid atherosclerosis (CIMT ≥ 1.1 mm) among euthyroid individuals, defined as having free triiodothyronine (free T3), free T4, and TSH levels with the normal range [3]. Therefore, independent of thyroid function, high–normal TPO-Ab titers might lead to the development of carotid atherosclerosis.

Atherosclerosis based on CIMT (mm) is an established risk factor for cardiovascular disease [4,5,6]. However, a previous study of 36,984 participants reported no significant associations between yearly CIMT progression (mm/year) and cardiovascular events [7]. Active arterial wall thickening (CIMT ≥ 0.01 mm/year), which indicates the status of yearly progression of CIMT, is inversely associated with baseline atherosclerosis (CIMT ≥ 1.1 mm), possibly due to the consumption of hematopoietic stem cells (CD34-positive cells) [8]. CD34-positive cells are known contributors to atherosclerosis; they have been reported to differentiate into macrophages and foam cells [9].

Since CIMT progression involves the process of endothelial repair, participants with baseline atherosclerosis might have lower endothelial repair activity, resulting in a lower chance of having active arterial wall thickening. Thus, we hypothesized that having a TPO-Ab titer in the normal range is positively associated with baseline atherosclerosis and inversely associated with active arterial wall thickening among euthyroid individuals.

To evaluate this hypothesis, we conducted a prospective study with three years of follow-up (2.8 ± 0.5) of 1069 Japanese individuals aged 40–74 years who participated in an annual health examination in 2014 and had free T3 and free T4 levels in the normal range and negative TPO-Ab titers.

## 2. Materials and Methods

### 2.1. Study Population

**Figure 1:**
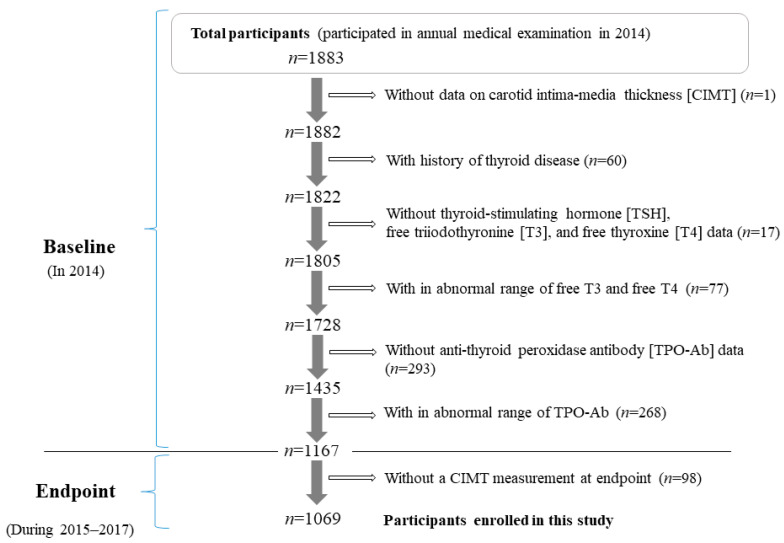
Demographics of study population.

Figure 1 shows the demographics of the study population. The methods related to thyroid function in the present risk survey have been described elsewhere [3]. The study population comprised 1883 Japanese individuals between the ages of 40 and 74 years from Saza town in western Japan who underwent an annual medical examination in 2014.

One patient without CIMT data (*n* = 1) was excluded from the present study. To avoid the influence of thyroid disease, participants with a history of thyroid disease (*n* = 60); participants without thyroid function data on TSH, free T3, and free T4 (*n* = 17); and participants with abnormal free T3 (normal range: 2.1–4.1 pg/mL) and free T4 (normal range: 1.0–1.7 ng/dL) levels were excluded (*n* = 77). In addition, participants without TPO-Ab data (*n* = 293) or abnormal TPO-Ab titers (normal range: <16 IU/mL) (*n* = 268) were excluded.

Participants who did not undergo an annual medical examination during the follow-up period, 2015–2017 (*n* = 98) were also excluded. A total of 1069 participants with a mean age of 61.0 years (standard deviation (SD): 8.8 years; range 40–74 years) were included in the study. The follow-up period of this study was 2.8 ± 0.5 years.

Informed consent was obtained from all study participants. This study was approved by the ethics committee of the Nagasaki University Graduate School of Biomedical Sciences (project registration number 14051404). All procedures involving human participants in this study were performed in accordance with the ethical standards of the institutional research committee and the 1964 Helsinki Declaration and its later amendments for comparable ethical standards.

### 2.2. Data Collection and Laboratory Measurement

Trained interviewers obtained information on clinical characteristics (history of thyroid disease). A fasting blood sample was collected. TSH, free T3, and free T4 levels were measured with chemiluminescent immunoassays at the LSI Medience Corporation (Tokyo, Japan). The normal range for free T3 (2.1–4.1 pg/mL), free T4 (1.0–1.7 ng/dL), and TSH (0.39–4.01 μIU/mL) based on this method were described elsewhere [10]. TPO-Ab titers were measured using standard procedures (electro chemiluminescence immunoassay) at the LSI Medience Corporation; the normal range (negative) was <16 IU/mL [10].

An experienced vascular examiner evaluated CIMT of both common carotid arteries using ultrasound inspection equipment: LOGIQ Book XP with a 10-MHz transducer (GE Healthcare, Milwaukee, WI, USA). Maximum values of CIMT in the common carotid arteries were calculated using semi-automated digital edge-detection software (Intimascope; MediaCross, Tokyo, Japan) with a previously described protocol [11]. Semi-automatically, this software recognized the edges of the internal and external membranes of the artery and determined the distance at a sub-pixel level (estimated to be 0.01 mm) [12]. We defined active arterial wall thickening as a CIMT increase of ≥0.01 mm/year, as in our previous study [8]. Furthermore, baseline atherosclerosis was diagnosed as CIMT ≥ 1.1 mm because a normal CIMT value was reported as <1.1 mm in a previous study [13]. The respective intra-observer variations for CIMT, which were assessed by two examiners, were simple correlation coefficients (r) = 0.91 (*p* < 0.01) and r = 0.89 (*p* < 0.001), and the inter-observer variation was r = 0.76 (*p* < 0.001).

### 2.3. Statistical Analysis

Characteristics of the study population were expressed as means ± SD except for gender and TSH. Gender was expressed as a percentage. Since the distribution of TSH was skewed, values were expressed as medians (interquartile range). Differences among various variables by sex-specific TPO-Ab tertile in the normal range (negative) were evaluated. Significant differences were evaluated using Student’s *t*-test for continuous variables and the χ^2^ test for categorical variables.

Logistic regression was used to calculate odds ratios (ORs) and 95% confidence intervals (CIs) to determine associations between baseline atherosclerosis and active arterial wall thickening, TPO-Ab titers in the normal range (negative) and baseline atherosclerosis, and TPO-Ab titers in the normal range (negative) and active arterial wall thickening.

In the analysis of the association between baseline atherosclerosis and active arterial wall thickening and the association between TPO-Ab titers in the normal range (negative) and baseline atherosclerosis, two models were used. Model 1 only adjusted for age and sex. Model 2 included variables in Model 1 plus potential confounding factors directly associated with thyroid function, namely, levels of TSH (μIU/mL) and free T3 (pg/mL).

In the analysis of the association between TPO-Ab titers in the normal range (negative) and active arterial wall thickening, three models were used. Model 1 only adjusted for age and sex. Model 2 included variables in Model 1 and levels of TSH (μIU/mL) and free T3 (pg/mL). Model 3 included variables in Model 2 plus baseline CIMT (mm). We performed a sex-specific analysis as a sensitivity analysis.

The goodness of fit of all logistic regression models in the present study was assessed using the Hosmer–Lemeshow test. All statistical analyses were performed with SAS for Windows (version 9.4: SAS Inc., Cary, NC, USA). Values of *p* < 0.05 were regarded as statistically significant.

## 3. Results

Among the present study population (*n* = 1069), 107 (10.0%) participants were diagnosed as having atherosclerosis at baseline, and 433 (40.5%) were found to have active arterial wall thickening during follow-up.

### 3.1. Characteristics of the Study Population

Characteristics of the study population by TPO-Ab tertile in the normal range (negative) are shown in Table 1. Having a normal TPO-Ab titer tertile was significantly positively associated with baseline CIMT but not with thyroid function (free T3, free T4, and TSH).

### 3.2. Association between Baseline Atherosclerosis and Active Arterial Wall Thickening

Table 2 shows the ORs and 95% CIs of baseline atherosclerosis for active arterial wall thickening. Baseline atherosclerosis is significantly inversely associated with active arterial wall thickening. This association was unchanged even after adjusting for thyroid function.

### 3.3. Association between TPO-Ab Titers in the Normal Range (Negative) and the Outcomes of Baseline Atherosclerosis and Active Arterial Wall Thickening

Table 3 shows the associations between TPO-Ab titers in the normal range (negative) and baseline atherosclerosis and between TPO-Ab titers in the normal range (negative) and active arterial wall thickening. Having a TPO-Ab titer in the normal range was significantly positively associated with baseline atherosclerosis. Having a TPO-Ab titer in the normal range was significantly inversely associated with active arterial wall thickening.

Table 4 shows the association between TPO-Ab titers in the normal range (negative) and baseline atherosclerosis and between TPO-Ab titers in the normal range (negative) and active arterial wall thickening in participants with TSH values within the normal range. We found essentially the same associations. Independent of known confounding factors, having a TPO-Ab titer in the normal range was significantly positively associated with baseline atherosclerosis and inversely associated with active arterial wall thickening.

### 3.4. Sensitivity Analysis

For a sensitivity analysis, we performed a sex-specific analysis for the association between TPO-Ab titers in the normal range (negative) and baseline atherosclerosis and for the association between TPO-Ab titers in the normal range (negative) and active arterial wall thickening. We found essentially the same associations. Among participants with TSH values within the normal range, the age-adjusted ORs (95% CIs) of logarithmic values of TPO-Ab titers for baseline atherosclerosis and active arterial wall thickening were 3.64 (1.30, 10.18) and 0.57 (0.32, 1.00) for males (*n* = 373) and 1.82 (0.63, 5.21) and 0.40 (0.19, 0.82) for females (*n* = 626), respectively.

## 4. Discussion

The main findings of the present study are that having a TPO-Ab titer in the normal range (negative) is positively associated with baseline atherosclerosis but inversely associated with active arterial wall thickening among euthyroid individuals. These findings are consistent with those of our previous cross-sectional study, which found an independent positive association between TPO-Ab titers in the normal range (negative) and atherosclerosis among euthyroid individuals [3].

Subclinical hypothyroidism is reported to be positively associated with carotid atherosclerosis [2]. Thus, a reduction in thyroid function could not explain the present results because similar associations were observed when we limited the analysis to participants with TSH levels within the normal range.

Inflammation is a known cause of endothelial dysfunction, which is related to atherosclerosis [14]. A previous case–control study of 1402 non-obese euthyroid individuals with autoimmune thyroiditis and 4206 non-obese euthyroid healthy controls found that thyroid function is not associated with inflammatory markers while TPO-Ab titers are positively associated with levels of high-sensitivity C-reactive protein [15]. Therefore, low-grade inflammation might be underlying the positive association between having a TPO-Ab titer in the normal range and baseline atherosclerosis in our present study.

However, in the present study, we also found a significant inverse association between TPO-Ab titers in the normal range (negative) and active arterial wall thickening among the euthyroid population.

Our previous prospective study of 363 Japanese males aged 60–69 years followed for two years revealed an inverse association between baseline atherosclerosis (CIMT ≥ 1.1 mm) and active arterial wall thickening (CIMT ≥ 0.01 mm/year), possibly due to lower levels of CD34-positive cells [8]. Active arterial wall thickening is a process of aggressive endothelial repair. During aggressive endothelial repair, many CD34-positive cells differentiate into mature (CD34-negative) cells such as macrophages and foam cells [9]. Progression of atherosclerotic lesions might reduce the number of circulating CD34-positive cells due to consumption. Therefore, participants with baseline atherosclerosis might have fewer CD34-positive cells and less active arterial wall thickening. In the present study, we found a significant inverse association between baseline atherosclerosis and active arterial wall thickening, as found in our previous study [8]. Participants with low levels of circulating CD34-positive cells might have a higher risk of cardiovascular disease than those with high levels of circulating CD34-positive cells [16,17] because low levels of CD34-positive cells indicate a deficiency in endothelial repair. Among patients with a history of atherothrombotic cerebral ischemic events, a strong inverse association between circulating CD34-positive cells and the number of cerebral infarcts was reported. However, this study did not find any correlation between the degrees of atherosclerosis and circulating CD34-positive cells [16].

The findings of the present study have many clinical implications. First, among euthyroid individuals, participants with high–normal TPO-Ab titers have a risk of developing atherosclerosis, as evaluated by CIMT. High–normal TPO-Ab titers could be associated with endothelial repair deficiency, which inhibits yearly increases in CIMT. This study also indicates that not having yearly CIMT progression does not always indicate a healthy endothelium.

Potential limitations of the present study warrant consideration. Although endothelial repair deficiency might be underlying the inverse association between having a TPO-Ab titer in the normal range and active arterial wall thickening, we could not evaluate the degree of endothelial deficiency directly. Since fewer circulating CD34-positive cells could indicate endothelial deficiency directly [8,18], further studies with data on circulating CD34-positive cell counts are necessary. Low-grade inflammation also might be underlying the present associations, but we could not evaluate inflammation directly.

## 5. Conclusions

In conclusion, among euthyroid individuals, having a TPO-Ab titer within the normal range (negative) is positively associated with baseline atherosclerosis but inversely associated with active arterial wall thickening. These findings can help clarify the mechanisms of endothelial maintenance.

## Figures and Tables

**Table 1:** Characteristics of study population by anti-thyroid peroxidase antibody (TPO-Ab) titer levels in the normal range (negative).

	TPO-Ab Tertile in the Normal Range (Negative)			*p*
	Low–Normal (*n* = 329)	Medium (*n* = 421)	High–Normal (*n* = 319)	
Male, %	49.5	25.2	42.3	<0.001
Age, years	61.3 ± 8.7	60.7 ± 9.0	61.2 ± 8.8	0.585
TSH, (0.39–4.01) μIU/mL	1.58 (1.10, 2.33) *^1^	1.53 (1.09, 2.20) *^1^	1.52 (1.09, 2.16) *^1^	0.808 *^2^
Free T3, (2.1–4.1) pg/mL	3.2 ± 0.3	3.1 ± 0.3	3.2 ± 0.3	0.014
Free T4, (1.0–1.7) ng/dL	1.3 ± 0.2	1.2 ± 0.2	1.2 ± 0.2	0.135
Baseline CIMT, mm	0.87 ± 0.30	0.89 ± 0.60	1.14 ± 2.48	0.027

TSH, thyroid-stimulating hormone; free T3, free triiodothyronine; free T4, free thyroxine; CIMT, carotid intima-media thickness; TPO-Ab, anti-thyroid peroxidase antibody. Values are means ± standard deviation unless otherwise stated. *^1^: Values are medians (interquartile range). *^2^: Logarithmic transformation was performed. The normal range is given in parentheses.

**Table 2:** Odds ratios (ORs) and 95% confidence intervals (CIs) for active arterial wall thickening by baseline atherosclerosis status.

	Baseline Atherosclerosis		*p*	*p* (Goodness-of-Fit Test)
	Absent (*n* = 962)	Present (*n* = 107)		
No. of cases (%)	420 (43.7)	13 (12.1)		
Model 1	Ref	0.16 (0.09, 0.29)	<0.001	0.601
Model 2	Ref	0.16 (0.09, 0.29)	<0.001	0.265

Ref, referent. Model 1 adjusted for age and sex. Model 2 adjusted for age, sex, and levels of free triiodothyronine (free T3) and thyroid-stimulating hormone (TSH).

**Table 3:** Odds ratios (ORs) and 95% confidence intervals (CIs) of anti-thyroid peroxidase antibody (TPO-Ab) titer in the normal range (negative) for baseline atherosclerosis and active arterial wall thickening.

	TPO-Ab Tertile in the Normal Range (Negative)			*p*	Log (TPO-Ab)	*p* (Goodness-of-Fit Test) *
	Low–Normal (*n* = 329)	Medium (*n* = 421)	High–Normal (*n* = 319)			
**Baseline atherosclerosis**						
No. of cases (%)	31 (9.4)	34 (8.1)	42 (13.2)			
Model 1	Ref	0.98 (0.58, 1.66)	1.52 (0.92, 2.51)	0.091	2.17 (1.08, 4.37)	0.729
Model 2	Ref	0.98 (0.58, 1.66)	1.52 (0.92, 2.50)	0.095	2.16 (1.07, 4.35)	0.222
**Active arterial wall thickening**						
No. of cases (%)	153 (46.5)	167 (39.7)	113 (35.4)			
Model 1	Ref	0.78 (0.58, 1.05)	0.64 (0.46, 0.87)	0.005	0.51 (0.33, 0.78)	0.093
Model 2	Ref	0.77 (0.57, 1.04)	0.63 (0.46, 0.86)	0.004	0.50 (0.33, 0.77)	0.421
Model 3	Ref	0.75 (0.55, 1.04)	0.70 (0.50, 0.97)	0.041	0.59 (0.37, 0.93)	0.243

Ref, referent. * Calculated for the association between logarithmic values of anti-thyroid peroxidase antibody (TPO-Ab) titer and outcome. Model 1 adjusted for age and sex. Model 2 adjusted for age, sex, and levels of free triiodothyronine (free T3) and thyroid-stimulating hormone (TSH). Model 3 adjusted for variables in Model 2 plus baseline carotid-intima media thickness (CIMT).

**Table 4:** Odds ratios (ORs) and 95% confidence intervals (CIs) of anti-thyroid peroxidase antibody (TPO-Ab) titer for baseline atherosclerosis and active arterial wall thickening among participants with thyroid-stimulating hormone (TSH) values in the normal range.

	TPO-Ab Tertile in the Normal Range (Negative)			*p*	Log (TPO-Ab)	*p* (Goodness-of-Fit Test) *
	Low–Normal (*n* = 301)	Medium (*n* = 394)	High–Normal (*n* = 304)			
**Baseline atherosclerosis**						
No. of cases (%)	26 (8.6)	33 (8.4)	39 (12.8)			
Model 1	Ref	1.10 (0.63, 1.92)	1.66 (0.98, 2.84)	0.055	2.58 (1.23, 5.40)	0.617
Model 2	Ref	1.09 (0.96, 2.80)	1.64 (0.96, 2.80)	0.063	2.54 (1.21, 5.34)	0.425
**Active arterial wall thickening**						
No. of cases (%)	144 (47.8)	155 (39.3)	111 (36.5)			
Model 1	Ref	0.73 (0.53, 0.99)	0.63 (0.46, 0.88)	0.006	0.50 (0.32, 0.78)	0.346
Model 2	Ref	0.72 (0.53, 0.99)	0.63 (0.46, 0.88)	0.006	0.50 (0.32, 0.78)	0.512
Model 3	Ref	0.70 (0.51, 0.98)	0.70 (0.49, 0.99)	0.044	0.59 (0.37, 0.94)	0.110

Ref, referent. * Calculated for the association between logarithmic values of anti-thyroid peroxidase antibody (TPO-Ab) titer and outcome. Model 1 adjusted for age and sex. Model 2 adjusted for age, sex, and levels of free triiodothyronine (free T3) and thyroid-stimulating hormone (TSH). Model 3 adjusted for variables in Model 2 plus baseline carotid-intima media thickness (CIMT).

## Data Availability

We cannot publicly provide individual data due to participant privacy according to the ethical guidelines in Japan. Additionally, the informed consent obtained does not include a provision for publicly sharing data. Qualifying researchers may apply to access a minimal dataset by contacting Prof Naomi Hayashida, Principal Investigator, Division of Promotion of Collaborative Research on Radiation and Environment Health Effects, Atomic Bomb Disease Institute, Nagasaki University, Nagasaki, Japan at naomin@nagasaki-u.ac.jp. Or, please contact the office of data management at ritouken@vc.fctv-net.jp. Information for where data can be requested is also available at https://www.genken.nagasaki-u.ac.jp/dscr/message/ and http://www.med.nagasaki-u.ac.jp/cm/ (accesed on 20 December 2021).
